# 2080 Implementing and evaluating an evidence-based intervention from the intensive care unit (ICU) setting into primary care using promotoras to reduce CA-MRSA recurrence and household transmission

**DOI:** 10.1017/cts.2018.255

**Published:** 2018-11-21

**Authors:** Brianna M. D’Orazio, Jonathan N. Tobin, Rhonda G. Kost, Chamanara Khalida, Jessica Ramachandran, Mina Pastagia, Teresa H. Evering, Maria P. de la Gandara, Cameron Coffran, Joel Correa da Rosa, Kimberly Vasquez, Getaw W. Hassen, Franco Barsanti, Satoko Kanahara, Regina Hammock, Rosalee Nguyen, Mark Trezia, Trang Gisler, Herminia de Lencastre, Alexander Tomasz, Barry S. Coller

**Affiliations:** 1 Clinical Directors Network (CDN); 2 The Rockefeller University; 3 Mount Sinai School of Medicine; 4 Metropolitan Hospital; 5 Urban Health Plan; 6 Community Healthcare Network; 7 Coney Island Hospital; 8 MyOwnMed, Inc.

## Abstract

OBJECTIVES/SPECIFIC AIMS: Community-associated methicillin-resistant *Staphylococcus aureus* (CA-MRSA) skin and soft tissue infections (SSTIs) recurrence ranges from 16% to 43% and presents significant challenges to clinicians, patients, and families. This comparative effectiveness research study aims to disseminate, implement and evaluate whether an existing intervention, consisting of decolonization and decontamination procedures, which has been determined to be effective in hospital intensive care unit settings, can be implemented by Community Health Workers (CHWs) or “promotoras” conducting home visits prevent recurrence of CA-MRSA and transmission within their households for patients presenting to primary care with SSTIs. METHODS/STUDY POPULATION: In partnership with 3 Community Health Centers and 4 community hospitals in NYC, this study will recruit patients (n=278) with confirmed MRSA SSTIs and their household members. Participants are randomized to receive either a CHW/Promotora-delivered decolonization-decontamination intervention or usual care, which includes hygiene education. The highly engaged stakeholder team meets monthly to review interim results, identify areas for refinement and new research questions, and develop and implement strategies to improve participant engagement and retention. RESULTS/ANTICIPATED RESULTS: MRSA and MSSA were found in 19% and 21.1% of wound cultures, respectively. 59.5% with MRSA+ wound culture had one or more MRSA+ surveillance culture; 67.8% with MSSA+ wound culture had one or more MSSA+ surveillance culture. The “warm handoff” approach, developed and implemented by the stakeholder team to engage patients from their initial consent to return of lab results and scheduling of the home visits, helped improve completion of baseline home visits by 14%, from 45% to 59% of eligible participants. Home visits have demonstrated that 60% of households had at least one surface contaminated with *S. aureus*. Of the surfaces that tested positive in the households, nearly 20% were MRSA and 81% were MSSA; 32.5% of household members had at least one surveillance culture positive for *S. aureus* (MRSA: 7.7%, MSSA: 92.3%). DISCUSSION/SIGNIFICANCE OF IMPACT: This study aims to understand the systems-level, patient-level, and environmental-level factors associated with SSTI recurrence and household transmission, and to examine the interactions between bacterial genotypic and clinical/phenotypic factors on decontamination, decolonization, SSTI recurrence and household transmission. This study will evaluate the barriers and facilitators of implementation of home visits by CHWs in underserved populations, and aims to strengthen the weak evidence base for implementation of strategies to reduce SSTI recurrence and household transmission.

